# Probiotic Therapy of Gastrointestinal Symptoms During COVID-19 Infection: A Randomized, Double-Blind, Placebo-Controlled, Remote Study

**DOI:** 10.3390/nu16223970

**Published:** 2024-11-20

**Authors:** Angela Horvath, Rosa Haller, Nicole Feldbacher, Hansjörg Habisch, Kristina Žukauskaitė, Tobias Madl, Vanessa Stadlbauer

**Affiliations:** 1 Division for Gastroenterology and Hepatology, Department of Internal Medicine, Medical University of Graz, 8010 Graz, Austria; angela.horvath@medunigraz.at.at (A.H.);; 2 Center for Biomarker Research in Medicine (CBmed), Division Translational Precision Medicine, 8010 Graz, Austria; 3 Otto Loewi Research Center, Medicinal Chemistry, Medical University of Graz, 8010 Graz, Austria; 4 Institute of Biosciences, Life Sciences Center, Vilnius University, 01513 Vilnius, Lithuania; 5 BioTechMed-Graz, 8010 Graz, Austria

**Keywords:** COVID-19, probiotic, microbiome, patient-reported outcomes, quality of life, *Enterococcus faecium*

## Abstract

Background: The novel coronavirus (SARS-CoV-2) led to gastrointestinal manifestations in up to 50% of cases, with diarrhea being common, and probiotics have been suggested as a potential treatment. Aim: This study aimed to assess changes in the microbiome and the effects of a multispecies probiotic in patients with COVID-19 in home quarantine through a fully remote telemedical approach. Methods: Thirty patients were randomized to receive either the Ecologic AAD probiotic (Winclove Probiotics, Amsterdam, The Netherlands), on the market as OMNi-BiOTiC 10 (Allergosan, Austria), or a placebo for 30 days in a 2:1 ratio. Respiratory and gastrointestinal symptoms were monitored in 2–10-day intervals via online surveys, and five stool samples were collected during the 30-day study period for microbiome and metabolomics analyses. Twenty-four healthy volunteers served as controls. Results: Of the 30 patients, 26 completed this study (10 placebo, 16 probiotic). Patients reported respiratory symptoms and a diminished gastrointestinal quality of life, both of which improved significantly during the study period, irrespective of the intervention. Compared to controls, infected patients showed significant alterations in the fecal microbiome (*p* = 0.002), including an increase in Bacteroidetes and decreases in Christensenellaceae, Ruminococcaceae, and Gammaproteobacteria, along with metabolomic changes. Probiotic treatment significantly modulated the patients’ microbiome beta diversity (*p* = 0.001) and introduced the *Enterococcus faecium* W54 strain. Symptoms, COVID-19-related taxa, and the fecal metabolome were not affected by the intervention. Conclusions: Patients with mild COVID-19 disease in home quarantine exhibited respiratory symptoms, a reduced gastrointestinal quality of life, and changes in the fecal microbiome and metabolome.

## 1. Introduction

The novel coronavirus (SARS-CoV-2) disease (COVID-19) outbreak began in Wuhan, Hubei province, in December 2019, spread throughout China in early 2020, and developed as a pandemic thereafter. Although the virus mainly causes respiratory symptoms, gastrointestinal symptoms have been reported in and outside of China [1,2]. Patients may present with anorexia, nausea, vomiting, diarrhea, and abdominal discomfort. Also, a fecal–oral transmission of the virus was suspected [3,4,5]. In patients with inflammatory bowel disease suffering from post-acute COVID-19 symptoms, viral DNA was present in intestinal tissue [6]. Clinical studies showed a variable incidence rate of diarrhea, ranging from 2 to 50% of cases; gastrointestinal symptoms may precede or follow respiratory symptoms [7]. SARS-CoV-2 uses the angiotensin-converting enzyme 2 (ACE2) for cellular entry. ACE2 is expressed in the small intestinal epithelia as well as in the upper esophagus, liver, and colon [7]. Diarrhea, which is usually mild, is associated with prolonged symptoms and viral carriage [8]. Fecal calprotectin and neopterin were elevated in stool of patients with COVID-19 diarrhea, indicating a relationship between gastrointestinal symptoms and this well-established biomarker of intestinal inflammation [9,10]. However, other studies showed a reduced inflammatory reaction in patients with gastrointestinal symptoms, which was also associated with reduced mortality [11]. While early reports from China suggested an increased mortality in patients with gastrointestinal symptoms, in meta-analyses, no clear association of gastrointestinal symptoms with mortality was shown [12,13].

The human gut microbiome composition is associated with COVID-19 disease severity and the immune response, which suggests a potential pathophysiological role [14]. Furthermore, the gut microbiome has been identified as a potential source of biomarkers and a therapeutic target [15,16,17,18]. Therefore, microbiome modulation, for example, by using prebiotics, probiotics, or synbiotics (a combination of probiotics with prebiotics), has been suggested in the management of COVID-19 [19,20]. China’s National Health Commission recommended the use of probiotics for the treatment of patients with severe COVID-19 in order to preserve intestinal balance and to prevent secondary bacterial infections, without any available clinical studies to support this [7], and probiotics apparently were used in Zhejiang during the COVID-19 pandemic [21]. A meta-analysis on the use of probiotics showed a significant improvement in headache, cough, and diarrhea [22]. A potential benefit of probiotics has also been described in meta-analyses for the treatment of upper respiratory tract infections, and viral gastroenteritis of other origins [23,24]. From a mechanistic point, *Enterococcus faecium* has been shown to have antiviral effects in enteropathogenic coronavirus-transmissible gastroenteritis virus infections in piglets [25]. Probiotics have been shown to improve symptoms and viral clearance [26] but in another study, no effect on perceived stress levels was observed [27]. Higher intakes of fermented foods showed an association with decreased mortality from COVID-19 [28]. We therefore aimed to characterize changes in the microbiome of patients with mild COVID-19 infection in home quarantine and assess the role of probiotics in the therapy of COVID-19 infection with gastrointestinal symptoms in a randomized, placebo-controlled, fully remote study.

## 2. Materials and Methods

### 2.1. Study Design

We performed a randomized, double-blind, placebo-controlled, fully remote study in patients with acute, non-severe COVID-19 disease (i.e., patients with positive PCR test without need of hospitalization). The study protocol was approved by the institutional review board (32-513 ex 19/20), and the study was registered at https://clinicaltrials.gov/ (NCT04420676, accessed on 16 November 2024).

### 2.2. Participants

Patients were recruited via the “Probando” and “Studienteilnehmergesucht.at” platforms and by the study group using mailings to all practicing physicians in the county of Styria, social media and newspaper advertisements, mass mailing in areas with high COVID-19 incidence, and flyers distributed in supermarkets, doctors’ offices, and pharmacies in Austria. Interested patients then called the study team or registered at a web-based platform. Additionally, stool samples of 24 healthy controls (age above 18 years, no acute or chronic disease, no intake of probiotics or antibiotics within the last 4 weeks, no recent COVID-19 infection) were collected during the pandemic to serve as a reference for COVID-related changes in the microbiome. After agreeing verbally to a screening interview or signing a general online informed consent, patients were screened with a standardized questionnaire. When they fulfilled the inclusion criteria and did not fulfill any exclusion criterion, they were asked to participate in this study. The informed consent procedure for this study was performed verbally via a video conference and the signed document was sent by post to the study center. After informed consent, patients were randomized and the study medication and the stool sampling kit were express shipped to the patients.

### 2.3. In- and Exclusion Criteria

Patients were included if they were 18 years or older, had an acute COVID-19 infection diagnosed by a positive SARS-CoV-2 PCR result less than 10 days ago (from a nasopharyngeal swab), and gave informed (tele)consent. Exclusion criteria were pre-existing diarrhea (including but not restricted to chronic inflammatory bowel disease, chronic diarrhea of other causes, and acute diarrheal illness within the 4 weeks before inclusion), antibiotic therapy 4 to 1 week before inclusion, probiotic treatment within the 4 weeks before inclusion, and technical difficulties in performing remote study visits.

### 2.4. Intervention and Randomization

After fulfilling all inclusion criteria and none of the exclusion criteria, patients were randomized into 2 groups in a 2:1 ratio. Group 1 received a probiotic mixture Ecologic AAD (Winclove Probiotics, The Netherlands), on the market as OMNi-BiOTiC 10 (Allergosan, Graz, Austria), twice a day for 30 days. Group 2 received a similar looking and tasting placebo without bacteria, twice a day. The products were dispensed in blank sachets in consecutively numbered cardboard boxes to ensure the blinding of participants, care takers, investigators, and outcome assessors. The study product Ecologic AAD (Winclove Probiotics, Amsterdam, The Netherlands), on the market as OMNi-BiOTiC 10 (Allergosan, Austria), contains *Bifidobacterium bifidum* W23, *Bifidobacterium lactis* W51, *Enterococcus faecium* W54, *Lactobacillus acidophilus* W37, *Lactobacillus acidophilus* W55, *Lactocaseibacillus paracasei* W20 (formerly known as *Lactobacillus paracasei* W20), *Lactoplantibacillus plantarum* W1 (formerly known as *Lactobacillus plantarum* W1), Lactoplantibacillus plantarum W62 (formerly known as *Lactobacillus plantarum* W62), *Lactocaseibacillus rhamnosus* W71 (formerly known as *Lactobacillus rhamnosus* W71), and *Ligilactobacillus salivarius* W24 (formerly known as *Lactobacillus salivarius* W24), which are embedded in a matrix containing maize starch, maltodextrin, inulin, potassium chloride, hydrolyzed rice protein, magnesium sulfate, fructooligosaccharide (FOS), enzymes (amylases), vanilla flavor, and manganese sulfate. The product has been chosen because of its beneficial effects on the gut microbiome and the clinical capacity to reduce diarrhea [29,30,31]. The dispensed probiotic supplement contains a mixture of the 10 above-mentioned strains with at least 5 billion organisms per 1 portion (=5 g). 

Randomization was carried out using the “Randomizer” software Version 2.2.0 (Institute of Medical Informatics, Medical University of Graz, Graz, Austria, accessed on 16 November 2024). Patients, caregivers, investigators, and outcome assessors were blinded to the allocation. The study product was packaged in plain sequentially numbered sachets.

### 2.5. Outcomes

Once the patients were recruited for this study and agreed to participate, they were asked to report their symptoms at baseline and every other day until day 10, every 5 days until day 20, and on day 30 via a structured telephone or online questionnaire, organized over the online survey resource “SociSurvey” (https://www.soscisurvey.de/, accessed on 16 November 2024). On day 0, day 5, day 10, day 20, and day 30, patients were asked to collect a stool sample in a storage tube. At the end of the study, patients were asked to return unused study medication, the study diary, as well as the stool samples.

The primary endpoint of the study was improvement in stool calprotectin; secondary endpoints were duration of diarrhea (defined as days with 3 or more loose stools), stool frequency (evacuations per days), stool consistency (according to the Bristol stool scale for each evacuation), other gastrointestinal symptoms (anorexia, nausea, vomiting, abdominal pain, bloating), duration of COVID-19 disease, severity of COVID-19 disease, stool microbiome composition, and adverse events.

Diarrhea was quantified using the Bristol stool scale [32] and a stool frequency chart where patients indicated the number and consistency of stools. Diarrhea was defined as three or more loose stools per day. Other gastrointestinal symptoms were assessed by the Gastrointestinal Quality of Life Index (GIQLI). The questionnaire consists of 36 questions each with 5 response categories and is validated in German [33].

The disease severity of COVID-19 disease was assessed by the number of days patients felt sick quantified by a translated version of the Acute Respiratory Tract Infection Questionnaire (ARTIQ) [34], body temperature curve, the number of days that patients were on sick leave (or equivalent if they were not working), the rate of hospitalizations, and, in case of hospitalization, the need for oxygen, the need for mechanical ventilation, and the duration of hospital stay, as well as the outcome (discharge, transfer to another health care facility, death).

Stool was collected by the patients at home in fecal sample collection tubes with a stabilizer (DNA/RNA Shield Fecal collection tube, ZymoResearch, Irvine, CA, USA). Unfortunately, contrary to the information provided by the company, it was not possible to assess calprotectin in stool with this preservation solution (see Supplementary Materials, Figure S1).

This study adhered to the CONSORT reporting guidelines to ensure transparency and rigor in the research methodology.

### 2.6. Sample Size Calculation

Sample size calculation was based on stool calprotectin. Stool calprotectin in COVID-19 patients with intestinal inflammation was on average 116 ± 183 µg/g and 16.8 µg/g in patients without intestinal inflammation. Means and standard deviations were estimated from medians and IQRs given in the publication using the method by Luo et al. [35,36]. With an alpha of 0.05, a beta of 80%, and an expected dropout rate of 20%, and a 1:2 randomization ratio, we estimated that we would need 120 patients to prove this hypothesis (performed with https://clincalc.com/, accessed on 16 November 2024).

### 2.7. Outcome Assessements

#### 2.7.1. Microbiome Analysis

DNA from stool samples was extracted using the QIAamp Fast DNA stool mini kit (QIAGEN, Hilden, Germany) automated on the QIAcube (QIAGEN, Hilden, Germany). Stool was transferred to 0.70 mm Garnet Bead tubes filled with 1 mL InhibitEx buffer (QIAGEN, Hilden, Germany). Subsequently, bead beating was performed using a SpeedMill PLUS (Analytik Jena, Jena, Germany) for 45 s at 60 Hz. Samples were then heated to 95 °C for 5 min and centrifuged for 1 min at 10,000 rpm. A total of 200 µL of the resulting supernatant was transferred to a 1.5 mL microcentrifuge tube, which was placed in the QIAcube for follow-up automated DNA isolation according to the manufacturer’s protocol. DNA was eluted from the QIAamp silica gel membrane with 200 µL TE buffer.

Isolated DNA was used to amplify hypervariable regions V1 and V2 of the *16S rRNA* gene using the primer pair 27F-338R in a dual-barcoding approach according to Caporaso et al. [37]. A total of 3 µL DNA was used for amplification and PCR products were verified using electrophoresis in agarose gel. PCR products were normalized using the SequalPrep Normalization Plate Kit (Thermo Fischer Scientific, Waltham, MA, USA), pooled equimolarily, and sequenced on the Illumina MiSeq v3 2 × 300 bp (Illumina Inc., San Diego, CA, USA). Demultiplexing after sequencing was based on 0 mismatches in the barcode sequences.

The resulting fastq files were used for bioinformatic preprocessing using QIIME2 tools V2023.2, which were implemented on a local Galaxy instance (https://galaxy.medunigraz.at/, 16 November 2024). Amplicon Sequencing variants (ASVs) were identified using the dada2 inference algorithm; taxonomic assignment was performed with a naïve Bayesian classifier trained on the SILVA database V132 with release at 99% identity. Cyanobacteria were regarded as possible contaminations and were therefore removed from the data set. On average, 27,774 ± 11,984 raw reads per sample were available for analysis, of which 16,556 ± 8423 high-quality reads per sample could be retained during quality filtering. The minimum high-quality read count and therefore the chosen rarefaction depth was 8002 reads. Read counts for all groups and timepoint are given in Table S1.

#### 2.7.2. Stool Metabolomics Analysis

Fecal samples were prepared for NMR spectroscopy measurements as previously described [38]. Briefly, 200 µL of samples (50 to 100 mg of stool in 2 mL of DNA/RNA Shield, ZymoResearch, Irvine, CA, USA) was mixed with 400 µL methanol in order to inactivate and precipitate proteins. The remaining solids were lysed using a Precellys homogenizer (Bertin Technologies SAS, Montigny-le-Bretonneux, France) and stored at −20 °C for 1 h, followed by centrifugation for 30 min at 10,000× *g* at 4 °C. Finally, the supernatants were lyophilized, resuspended in 500 μL NMR buffer (0.08 M Na_2_HPO_4_, 5 mM 3-(trimethylsilyl) propionic acid-2,2,3,3-d_4_ sodium salt (TSP), 0.04 (*w*/*v*) % NaN_3_ in D_2_O, with pH adjusted to 7.4 with HCl or NaOH, respectively), and transferred into 5 mm NMR tubes for measurement on the NMR instrument using the CPMG pulse sequence as described. Spectra preprocessing and data analysis were carried out using Matlab^®^ scripts (a group of Prof. Jeremy Nicholson at Imperial College London, London, UK). NMR data were imported to Matlab^®^ vR2014b (Mathworks, Natick, MA, USA); regions around the water, TSP, and remaining methanol signals were excluded, aligned, and corrected for sample metabolite dilution by probabilistic quotient normalization [39]. The reported values correspond to an arbitrary unit (A.U.) derived from the area under the peak being proportional to concentration.

### 2.8. Statistical Analysis

The primary endpoint (calprotectin in stool) could not be assessed due to technical problems (see Supplementary Materials). Secondary endpoints were assessed by Student’s *t*-test or the Mann–Whitney-u-test, depending on the distribution of the data, in the cross-sectional part of this study and (general) linear mixed-effect models for the interventional part of this study. The patients’ answers to GIQLI and ARTIQ were used to construct items and total scores according to published instructions. General mixed-effect models were used to analyze the influence of the intervention, time, and the interaction between intervention and time on the variation of the questionnaire responses. Analysis was performed in R version 4.2.2 (2022-10-31 ucrt) and the additional packages lme4, jtools, rcompanion, DescTools, tidyverse, readxl, and writexl.

For the statistical analysis of the microbiome data, count table, taxonomy, and sample data were handed off to the R package phyloseq. Alpha diversity was estimated from an even count table, rarefied to the minimum read count per sample, using the metrics observed OTUs, Shannon index, inverse Simpson (implemented in the phyloseq::estimate_richness() function), and Evenness (calculated as Shannon-index/log (observed OTUs). The influence of the probiotic intervention over time was assessed using a mixed-effect model (using the R package lme4) with the following structure: diversity_metric~Intervention * day+(1|Individual).

For beta diversity analysis, Bray–Curtis dissimilarity calculated with the phyloseq::ordinate() function was used as foundation for the distance matrix, and permutational multivariate analysis of variance using distance matrices (PERMANOVA), calculated using the vegan::adonis2() function, was used to determine whether there was significant clustering among the test groups. Principal coordinate analysis (PCoA) was used for low-dimensional visualization (phyloseq::plot_ordination()). Redundancy analysis (RDA) was performed on a Hellinger-transformed abundance table to determine influencing factors on the microbiome composition, using the vegan::rda() function. To describe changes in the microbiome in more detail, multivariate association analysis (MaAsLin2) was applied using a combination of the type of intervention and the day of intervention as a fixed effect and the individuals as a random effect to account for the dependence in the data. To identify differentially abundant taxa between patients and non-infected controls, the LDA effect size (LEfSe) implemented in the R package “Microbiomemarkers” was applied.

Predicted metagenomics were performed with the application TAX4FUN2 implemented on the web-based analysis platform “microbiome analyst” (https://www.microbiomeanalyst.ca/, Version 2.0, accessed on 21 March 2023) on a randomized count table with SILVA-based taxonomic annotations in order to estimate the functional potential of the studied microbiomes.

For the statistical analysis of stool metabolomics, we used the web-based analysis platform Metaboanalyst (https://www.metaboanalyst.ca/, accessed on 21 March 2023). The raw integral table was uploaded, metabolites were normalized to the sum of all metabolites in one sample, square root-transformed, mean-centered, and divided by the standard deviation. To determine the overall similarity of the stool microbiome of COVID-19 patients and non-infected controls and identify discriminant features, orthogonal partial least squares discriminant analysis (oPSL-DA) was used. For groupwise comparisons, a volcano plot based on t-tests and the Benjamani–Hochberg correction was created. To identify metabolites that were modulated by the probiotic intervention, linear mixed-effect models were applied as described above. In addition to the above-mentioned R packages, the following packages were used: ggpubr, ggplotify, ggConvexHull, ggrepel, ggh4x, and cowplot.

## 3. Results

### 3.1. Study Participants

**Figure 1:**
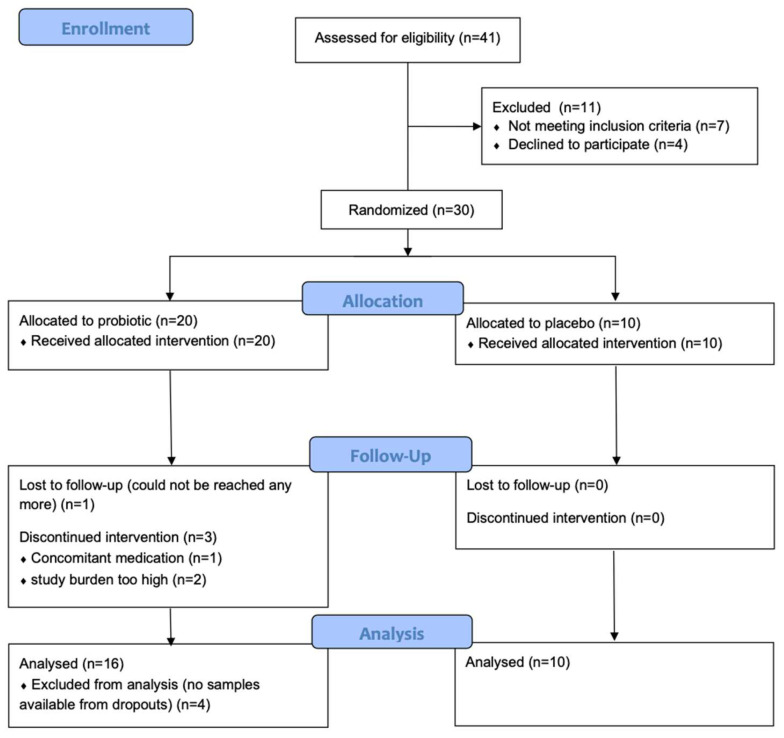
CONSORT flowchart of the study.

We started recruitment on 24.09.2020 and terminated this study on 1.12.2021. In total, 30 participants in home quarantine could be recruited. Twenty-six finished this study according to the protocol (see Figure 1). Ten of these patients were allocated to the placebo group, of whom six were female and four were male, with an average age of 34.1 ± 9.6 years, and sixteen patients were allocated to the probiotic group, ten female and six male, with an average age of 39.4 ± 13.1 years. Sex and age were also well matched between the treatment groups (*p* = 0.384 and *p* > 0.999, respectively). Patients in the probiotic group started the intervention on average 9 days and the patients in the placebo group 10 days after the positive PCR test (*p* = 0.579). No adverse events or severe adverse events attributable to the intervention were observed. For the comparison of microbiome and metabolome data, 24 healthy volunteers were recruited during the pandemic (18 female and 8 male, average age 37.9 ± 10.0); the controls were well matched for sex and age (*p* = 0.308 and *p* = 0.791).

### 3.2. COVID-19-Related Changes in the Intestinal Microbiome

**Figure 2:**
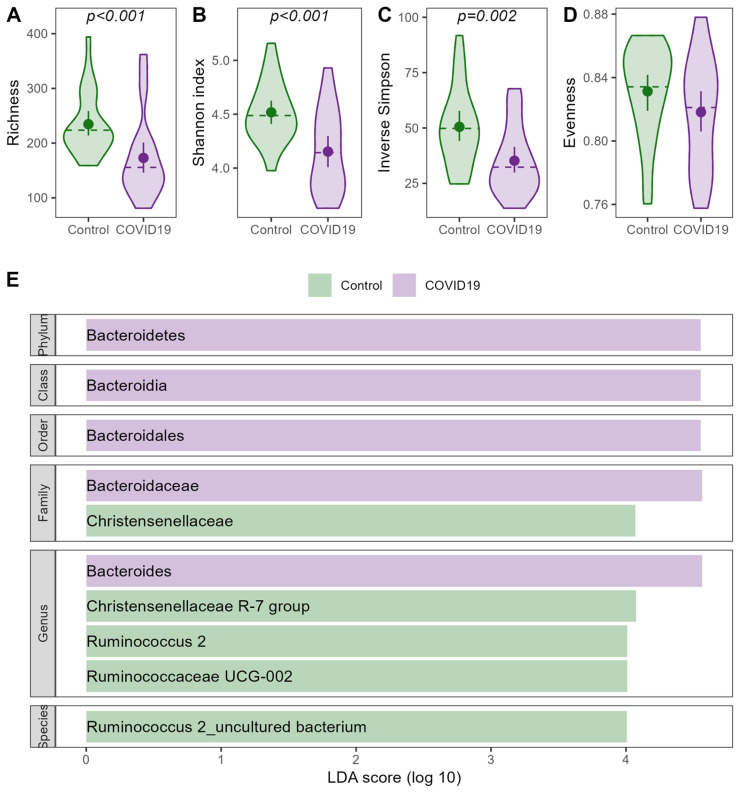
COVID-19-related changes in the intestinal microbiome. (**A**–**D**) Alpha diversity estimates for infected patients before intervention and non-infected controls. (**E**) LEfSe plot for differences in the intestinal microbiome between patients and controls.

The most common taxa on all taxonomic levels are visualized in Figure S2 and following. When compared to healthy controls, patients suffering from an acute COVID-19 infection showed significantly lower alpha diversity throughout the study period, while evenness did not differ between the groups (Figure 2). Beta diversity varied between patients and non-infected controls, but the infection explained only 2.7% of the observed variance (PERMANOVA: F = 1.321; R^2^ = 0.0268; *p* = 0.002). The differences in the microbiome composition were mainly driven by an increase in the Bacteroidetes taxa and a decrease in the *Christensenellaceae* and *Ruminococcaceae* taxa in the COVID-19-infected patients compared to healthy controls, as shown in Figure 2.

### 3.3. COVID-19-Related Changes in the Stool Metabolome

**Figure 3:**
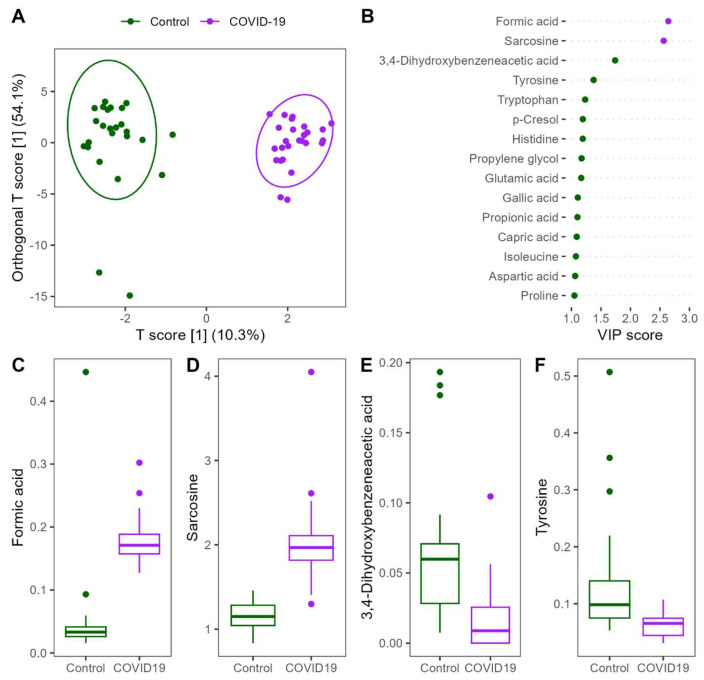
Changes in the stool metabolome of patients infected with COVID-19 compared to non-infected controls. (**A**) oPLS-DA plot showing clear separation between groups; (**B**) importance of the metabolites with the highest VIP scores. The color of the points indicates the group in which the metabolite was more concentrated. (**C**–**F**) Concentration of the top 4 metabolites in oPLS-DA.

Patients with COVID-19 showed significant alteration in the stool metabolome compared to non-infected controls. OPLS-DA showed a clear separation of the groups and identified formic acid, sarcosine, 3,4-dihydroxybenzeneacetic acid, and tyrosine as the most important discriminators. Formic acid and sarcosine were significantly higher in patients with COVID-19 infection, while 3,4-dihydroxybenzeneacetic acid and tyrosine were significantly lower in infected patients compared to non-infected controls (Figure 3).

### 3.4. Recovery and the Effects of the Probiotic Intervention

#### 3.4.1. Duration of Diarrhea, Stool Frequency, and Consistency

Clinically relevant diarrhea, defined as three or more loose stools per day, was rare throughout the study period. Only one patient in the placebo group recorded diarrhea on day seventeen of the intervention, and two patients in the probiotic group documented diarrhea. One patient recorded a single instance on day 2 of the intervention and a second patient reported it three days in a row, starting on day 13 of the intervention. Accordingly, few patients reported to be bothered by diarrhea (*n* = 4) or a frequent (*n* = 4) or urgent need for bowel movements (*n* = 3) in the GIQLI questionnaire all or most of the time. These numbers did not significantly change throughout the study period and were not influenced by the intervention. For details, see Figure S7.

#### 3.4.2. Other Gastrointestinal Symptoms

Gastrointestinal symptoms and their effect on the quality of life was systematically quantified by the GIQLI questionnaire. Gastrointestinal symptoms impaired quality of life at baseline but improved significantly over time (*p* = 0.006) irrespective of the intervention (*p* = 0.635). Similarly, the effect of gastrointestinal symptoms on the emotional well-being of the patients was more pronounced at the beginning of the study period and eased significantly throughout time (*p* = 0.009). The intervention did not influence the rate at which the patients recovered emotionally (*p* = 0.575). The same pattern was apparent in the recovery of physical and social functions over time (both *p* < 0.001), without significant influence of the intervention (*p* = 0.700 and *p* = 0.528, respectively). Treatment inconvenienced patients mainly in the first days of the study period, which improved significantly afterwards (*p* = 0.039); the intervention did not influence the rate of recovery (*p* = 0.329). Details are given in Figure S8.

#### 3.4.3. Duration and Severity of the COVID-19 Disease

**Figure 4:**
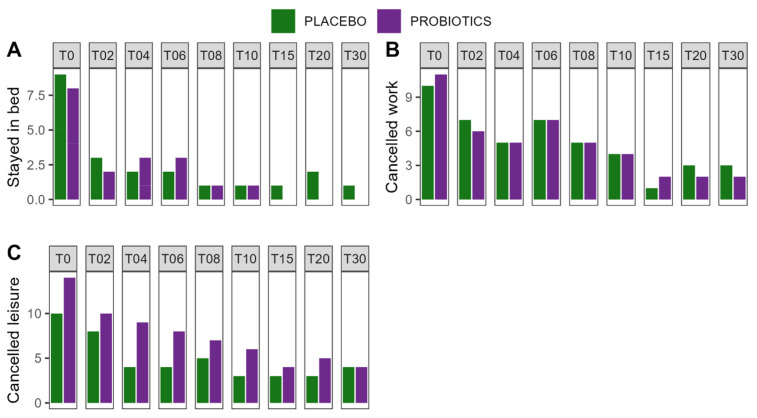
Recovery from COVID-19 disease. (**A**) Patients with the need to stay in bed because of COVID-19 disease; (**B**) patients with the need to cancel work activities because of COVID-19 disease; (**C**) patients with the need to cancel leisure activities because of COVID-19 disease. T—Timepoint of the study which corresponds to the day of intervention.

Acute symptoms of the COVID-19 disease were monitored by the Acute Respiratory Tract Infection Questionnaire (ARTIQ). Symptoms of the upper respiratory tract (*p* < 0.001), the lower respiratory tract (*p* < 0.001), psychological issues (*p* = 0.011), intake of medicine (*p* = 0.008), especially the use of antipyretics (*p* = 0.020), muscle pain (*p* = 0.001), and tiredness (*p* = 0.002) subsided significantly over time, irrespective of the intervention. Similarly, patients felt less need to stay in bed, cancel work, and cancel leisure activities over time, but recovery was not influenced by the intervention. Sleep was unaffected by time or the intervention. A summary of the results can be found in Figure 4 and Table S2. Reports of fever were rare; only two patients reported a body temperature of 38 °C or above, for one and two days, respectively.

**Figure 5:**
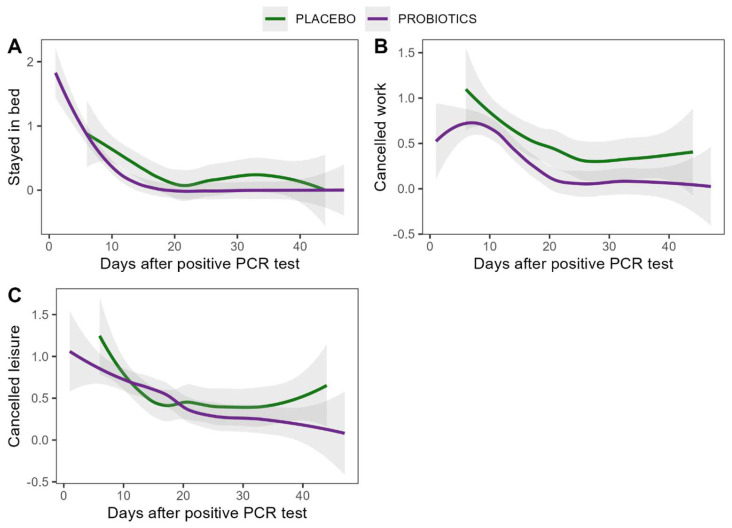
Recovery from COVID-19 disease as a function of days after positive PCR test for probiotic and placebo group. Models were fitted using Locally Weighted Scatterplot Smoothing (LOESS) regression. (**A**) Stayed in bed (**B**) Cancelled work (**C**) Cancelled leisure activities.

Time to recovery was comparable between treatment groups. Patients in the probiotic group started the intervention on average nine days after their positive PCR test and patients in the placebo group were one day further along in their infection course when they started the intervention. To account for this possible confounder, the course of recovery was corrected for the time since diagnosis. Both groups also showed comparable recovery rates after correction for time since the positive PCR test and an effect of the intervention was not detected, as shown in Figure 5.

#### 3.4.4. Stool Microbiome Composition and Metabolome

**Figure 6:**
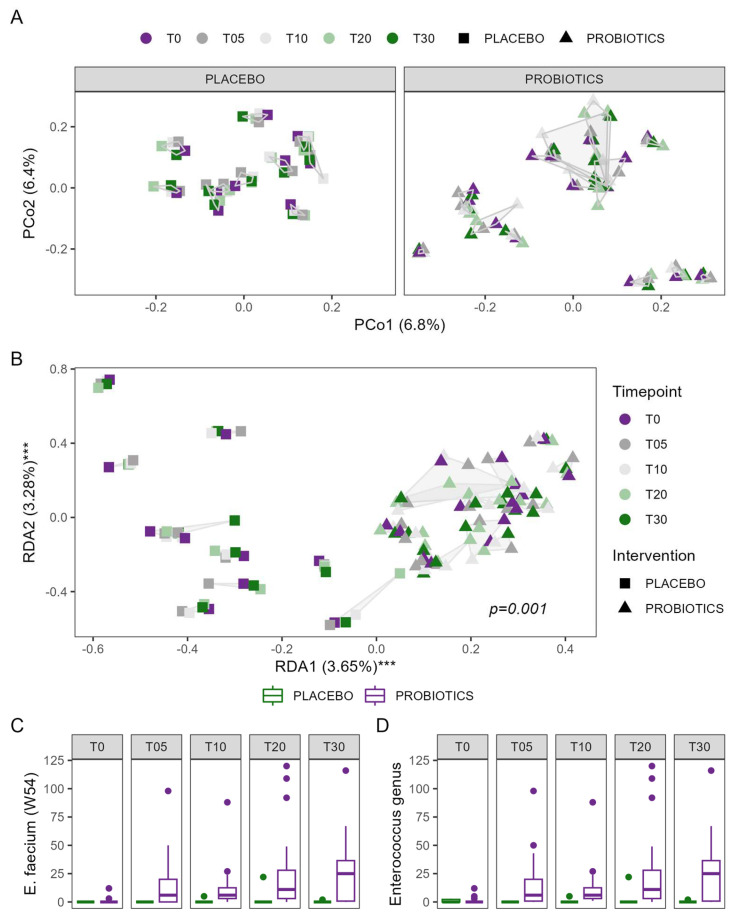
Changes in the microbiome composition of patients in the probiotic group compared to the placebo group; (**A**) PCoA plot of Bray–Curtis dissimilarities for the placebo group on the left side and the probiotic group on the right side; samples from the same individual are connected with a gray line; (**B**) visualization of the redundancy analysis testing the influence of intervention, time, and individual variation of the microbiome; *** *p* < 0.001; samples from the same individual are connected by a gray line; abundance of *Enterococcus faecium* (**C**) and its parent genus *Enterococcus* (**D**) throughout the study period in both groups.

Alpha diversity was comparable between patients allocated to the probiotic or placebo group and did not change significantly over time (Figure S9). Bray–Curtis dissimilarity was significantly influenced by the intervention over time, even though the effect was rather small (R^2^ = 0.002, F = 0.298, *p* = 0.017). Similar effects were observed for Jaccard (R^2^ = 0.003, F = 0.446, *p* = 0.002) and unweighted UniFrac (R^2^ = 0.004, F = 0.469, *p* = 0.049). Redundancy analysis confirmed that the intervention with the probiotic influenced the composition of the microbiome during this study (*p* = 0.001), as shown in Figure 6. Patients in the probiotic group showed the most pronounced changes in all dimensions and directions, and no clear directionality of probiotic-associated changes in the microbiome could be observed; see also Figure S10. Predicted metagenomics indicated no significant difference in the functional potential between the groups at baseline, which did not change during the intervention. The stool metabolome remained stable throughout the study in both groups and the probiotic intervention showed no significant effect on the metabolites.

In search of a clearer definition of the observed changes in the microbiome, multivariate association analysis identified a steady increase in *Enterococcus faecium*, one of the strains present in the administered study product, as well as its parent genus *Enterococcus*, in patients allocated to the probiotic group. The increase reached statistical significance on day 30, the last day of the probiotic intervention. In total, four ASVs of the genus *Enterococcus* were found across all samples. Two ASVs were identical to the Enterococcus faecium strain administered through the study product. Other enterococci were found very sparsely and with very low copy numbers per sample, irrespective of intervention or timepoint during the study; see also Figure S11. Interestingly, the other bacteria of the probiotic formulation were not as successfully recovered in the microbiome. While the *Lactobacillus acidophilus* strains (W37/W55, indistinguishable by *16S rRNA* gene sequencing) were recovered in 6 of the 129 samples (all in the probiotic group), the *Lactoplantibacillus plantarum* strains W1 and W62 in 2 samples each, and *Lactocaseibacillus paracasei* W20 in 1 sample of the probiotic group and 2 samples of the placebo group, the respective 16S sequences of strains W23, W51, W24, and W71 could not be found.

**Figure 7:**
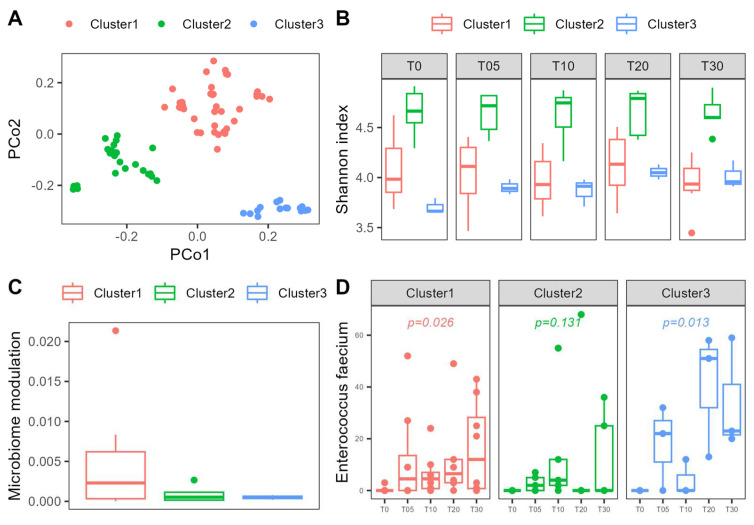
(**A**) Clustering of microbiome composition and the specific response to probiotic intervention. (**B**) Shannon index of patients of the probiotic group according to cluster allocation. (**C**) Microbiome modulation quantified as the area of the convex hull according to clustering. (**D**) Abundance of *Enterococcus faecium* as part of the probiotic formulation in different clusters.

**Figure 8:**
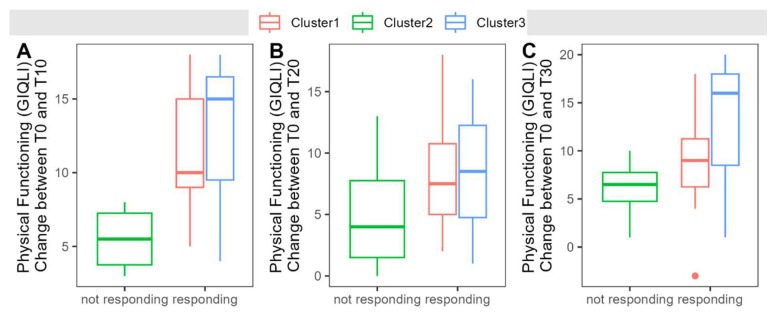
(**A**–**C**) Increase in physical functioning measured by the Gastrointestinal Quality of Life Index was more pronounced in responding clusters (i.e., clusters with stable probiotic enrichment) compared to the non-responding clusters.

Interestingly, the probiotic-treated patients show a distinct clustering into three clearly separated groups in the PCoA (Figure 7A). Patients in these clusters show comparable age and sex distribution, but significant differences in alpha diversity estimated by richness, the Shannon index, Inverse Simpson, and Chao1 index. Cluster 1 (n = 8) shows the strongest modulation of beta diversity during the intervention and a stable enrichment with probiotic bacteria. Alpha diversity is relatively low in this cluster and remains stable throughout the study period. Patients in cluster 2 (n = 5) are characterized by relatively high alpha diversity compared to the remaining clusters, but at the same time, no significant increase in *Enterococcus faecium* abundance during the intervention could be observed in this cluster. Cluster 3 (n = 3) shows little modulation of the microbiome during the intervention and a low alpha diversity but a significant increase in the Shannon index at timepoint T20 and T30. Cluster 1 shows significantly worse emotional well-being at the beginning of the study compared to Cluster 3 and significantly worse physical functioning (GIQLI) compared to Cluster 2. Clusters with stable probiotic enrichment (Cluster1 and Cluster3) show a steeper recovery in physical functioning (GIQLI), but this observation does not reach statistical significance (see also Figure 8). Other parameters do not show any differences between clusters. In regard to the modulation of the microbiome, Cluster 2 shows some fluctuation of an ASV from the genus *Escherichia-Shigella*, a temporary drop in an ASV from the genus *Ruminococcaceae UCG-014*, a steady decrease in a *Tyzzerella* ASV and a not further classified *Bacteroides* species, and undirected fluctuations of *Ruminococcaceae* and a *Christensenellaceae R7 group* ASV. Cluster 3 shows an increase in an ASV of the genus *Eggerthella* over time, a temporary drop in an ASV from the *Bacteroides gnavus* group, and a temporary surge in a *Butyricicoccus* ASV. Although the modulation is much more pronounced in Cluster 1, no commonly changed taxon could be identified. Correlation with the amount of modulation—defined as the area of the convex hull in the PCA plot—reveals sporadic changes in different ASVs in different patients.

## 4. Discussion

Our study clearly shows that even a non-severe acute COVID-19 infection alters microbiome composition and the fecal metabolome. In this randomized, double-blind, placebo-controlled, fully remote study in patients with acute, non-severe COVID-19 disease, we showed that the intake of a probiotic mixture led to a modulation of the microbiome over time, as well as good colonization with *Enterococcus faecium*.

In COVID-19, gut microbiome composition has been described to be significantly altered, with a decrease in gut microbiome diversity and an increase in opportunistic pathogens, indicating a loss in colonization resistance [40]. However, most studies so far have been performed in hospitalized patients with severe COVID-19 infection. Our study shows that even non-severe COVID-19 infections are associated with changes in the gut microbiome. This is evidenced by a lower alpha diversity and an altered beta diversity in our cohort and the observed taxonomic differences between patients and controls, namely an increase in the *Bacteroidetes* taxa and a decrease in the *Christensenellaceae* and *Ruminococcaceae* taxa. *Bacteroidetes* are producers of short-chain fatty acids and higher abundance is often associated with better health. On the other hand, a decreased abundance of *Christensenellaceae* has been observed in individuals with obesity and metabolic diseases, whereas reductions in *Ruminococcaceae* have been associated with several gastrointestinal disorders, such as inflammatory bowel diseases [41,42]. We also found clear differences in stool metabolome composition between patients with acute COVID-19 infection and controls, whereas no effect of the intervention could be observed. Formic acid and sarcosine were elevated whereas 3,4-dihydroxybenzeneacetic acid and tyrosine were decreased in patients with acute COVID-19. Formic acid contributes to the acidity of feces and is commonly used in livestock to optimize intestinal pH and improve intestinal digestibility and mineral utilization, as well as for its antimicrobial properties against specific pathogens [43]. An increase during an acute infection may therefore be interpreted as a reaction of the microbiome to prevent pathogen growth. Sarcosine is converted to glycine, which is important in many physiological functions, such as central nervous system homeostasis, and has been explored therapeutically in psychiatric diseases [44,45]. Tyrosine, as an amino acid precursor of neurotransmitter, has been associated with psychiatric and neurological disorders, metabolic diseases, and kidney injury [46,47,48]. 3,4-dihydroxybenzeneacetic acid is a microbial metabolite that is associated with health, especially with better quality of life and better mental health; therefore, a decrease in acute COVID-19 patients may be related to an impaired quality of life [49].

Probiotics were already used early on in the COVID-19 pandemic, first without solid evidence [7,21]. A recent large study [26] and a meta-analysis, which includes this study, showed a significant improvement in symptoms and viral clearance [22]. In another study, a spore-based probiotic showed a shorter time to resolution of symptoms in COVID-19-infected patients; however, the study was not randomized and probiotic intake was started before the infection occurred [50]. Probiotics also improved symptoms in other upper respiratory tract infections and viral gastroenteritis [23,24].

In our study, we observed an increased variability in the composition of the gut microbiome with the intake of the probiotic, which is encouraging, since it shows the potential of the probiotic to modulate microbiome composition. We observed three different clusters of patients in relation to their microbiome composition which showed differential response to probiotic treatment, indicating that the baseline microbiome composition of the host is relevant to their response to microbiome modulation treatment. Our data suggest that microbiomes with lower alpha diversity show better colonization with probiotic strains, potentially indicating a better response to probiotics. While this is encouraging for patients with diseases that are associated with low alpha diversity, it could also imply that it may be necessary to increase the dose or prolong the treatment duration in conditions with relatively high alpha diversity. We observed that of the 10 probiotic strains ingested with the study product, only *Enterococcus faecium* showed a significant, time-dependent increase in the microbiome. *Enterococcus faecium* has been shown to have antiviral effects in enteropathogenic coronavirus transmissible gastroenteritis virus infections in piglets [25] and against influenza A virus in vitro [25]. A probiotic *Enterococcus faecium* strain was shown to produce a bacteriocin which also exhibits antiviral activity [51]. Therefore, this strain is a promising choice for further mechanistic studies. In our study, intervention with a probiotic did not influence the reported symptoms or time to recovery, possibly due to the small sample size.

With this study, we prove the feasibility of performing fully remote clinical intervention trials during a pandemic. We also highlight the problem of many clinical trials—the difficulty to recruit an adequate number of participants. It is currently not known whether patients are more or less likely to participate in remotely conducted trials [52]. As in conventional study designs, remotely conducted studies also have difficulties in recruiting adequate sample sizes [53]. In our study, no other possibility than a fully remote study setup was available, since patients with non-severe acute COVID-19 infection were in quarantine at that time and could not visit the hospital for study purposes. A toolkit for studies during COVID-19 was published, indicating that creating awareness and opportunity is of utmost importance to achieve recruitment targets [54]. We put a considerable amount of effort into social media campaigns, mailings to physicians, flyer distribution, and conventional mailing; however, due to strict lockdown measures in Austria, it was impossible to follow suggestions such as direct contact with local organizations and companies such as shops, restaurants, or other frequented places because these places were closed and strict distance rules were in place. Despite these restrictive regulations and the overall cautious mindset of the Austrian population, we managed to include 30 patients in home quarantine during acute COVID-19 infection.

### Limitations

While the strength of our study is the fact that we were able to perform a randomized, placebo-controlled trial in a cohort that is not accessible with traditional study setups, the remote collection of biological samples of isolated individuals was clearly a challenge. On the one hand, contact with the outside world was restricted for patients and their entire household, and on the other hand, the fecal transmission of COVID-19 was discussed at the time of study [55], which made shipping and handling of fresh stool samples impossible at that time. Therefore, we decided to use a stabilizer solution to inactivate microorganisms, including viruses, which enabled patients to collect all samples at home, store them safely until the end of this study, and ship them back after home quarantine was lifted. Unexpectedly and contrary to the information provided by the company, the measurement of calprotectin, an emerging biomarker of intestinal inflammation in COVID-19 [56] and our predefined primary endpoint, was confounded by the stabilizer. Therefore, our study is limited to the secondary endpoints, microbiome composition, metabolomics, and patient-reported outcomes. The unusual circumstances of the pandemic also posed challenges to the patient recruitment process, which resulted in a small sample size. Nevertheless, this study contributes to a better understanding of the timing and direction of gut microbiome changes in acute viral infections.

## 5. Conclusions

In conclusion, we were able to show the feasibility of a fully remote study during pandemic conditions. We were further able to show that even a non-severe COVID-19 infection shows significant microbiome and fecal metabolome alterations and that the intake of a probiotic mixture during acute non-severe COVID-19 disease increases the individual variability of the microbiome.

## Data Availability

This study was registered at https://clinicaltrials.gov/ (NCT04420676, accessed on 16 November 2024). Sequencing data are available at the National Center for Biotechnology Information Sequence Read Archive (NCBI SRA, https://www.ncbi.nlm.nih.gov/sra, accessed on 16 November 2024) under the project accession number PRJNA1000423. The nuclear magnetic resonance (NMR) raw data have been deposited at MetaboLights under the accession number (the ID will be made available upon acceptance of the manuscript) (https://www.ebi.ac.uk/metabolights/, accessed on 16 November 2024). All other individual patient data are available upon reasonable request from the corresponding author.

## Supplementary Materials

The following Supporting Information can be downloaded at https://www.mdpi.com/article/10.3390/nu16223970/s1. Figure S1: Bland-Altman plot of differences between measurements of calprotectin in native stool and in stool collected in the stabilizer. Figure S2: Top 5 phyla observed in the microbiome; CTRL—uninfected controls, PLA—Placebo, PRB—Probiotics. Figure S3: Top 10 classes observed in the microbiome; CTRL—uninfected controls, PLA—Placebo, PRB—Probiotics. Figure S4: Top 10 orders observed in the microbiome; CTRL—uninfected controls, PLA—Placebo, PRB—Probiotics. Figure S5: Top 10 families observed in the microbiome; CTRL—uninfected controls, PLA—Placebo, PRB—Probiotics. Figure S6: Top 15 genera observed in the microbiome; CTRL—uninfected controls, PLA—Placebo, PRB—Probiotics. Figure S7: Results from the gastrointestinal quality of life index questions regarding diarrhea and the need for frequent or urgent bowel evacuations. Bar charts show the number of people answering the questions if they are inconvenienced by diarrhea (upper panel), frequent bowel movements (middle panel) or urgent bowel movements (lower panel) with “Yes, all the time” or “Yes, most of the time”, according to their group allocation. Figure S8: Development of the gastrointestinal quality of life during the study period in both treatment groups. Figure S9: Alpha diversity metrics of patients with COVID19-infection throughout the study period and compared to healthy controls. Figure S10: Similarity of the microbiome composition of healthy controls and patients with COVID19-infection before and at the end of intervention. Arrows represent the changes of a patient’s microbiome from the beginning (base) and the end (tip) of the intervention, purple represents the probiotic group, grey the placebo group and green represents the non-infected controls. Figure S11: Enterococci ASVs found throughout the study according to timepoint and group allocation. Unique ASVs are presented in different colors. Blue and green bars represent the ASVs that most likely originated from the probiotic bacterium in the study product. Table S1: Read counts before and after bioinformatics processing and quality filtering in all groups and at all timepoints. Table S2: Results of the mixed effect models testing the influence of time and intervention on the recovery from COVID-19 disease. Table S3: Patient reported outcomes for the intervention groups at all timepoints given as mean (sd).
