# Supplementary material for: Probiotic Therapy of Gastrointestinal Symptoms During COVID-19 Infection: A Randomized, Double-Blind, Placebo-Controlled, Remote Study
Source: Nutrients. 2024 Nov 20;16(22):3970. doi: 10.3390/nu16223970 (PMC11597392; doi:10.3390/nu16223970)
Supplement: Supplementary file 1 [file nutrients-16-03970-s001.zip › nutrients-3272255-supplementary.pdf]

Supplementary information

Probiotic therapy of gastrointestinal symptoms during COVID-19 infection: A randomized, double-blind, placebo controlled, remote study

Angela Horvath [1,2], Rosa Haller [1,2], Nicole Feldbacher [1,2], Hansjörg Habisch [3], Kristina Žukauskaitė [1,4], Tobias Madl [3,5], Vanessa Stadlbauer [1,2]

[1]Division for Gastroenterology and Hepatology, Department of Internal Medicine, Medical University of Graz, Graz, Austria;

[2]Center for Biomarker Research in Medicine (CBmed), Graz, Austria;

[3]Gottfried Schatz Research Center for Cell Signaling, Metabolism and Aging, Molecular Biology and Biochemistry, Medical University of Graz, Graz, Austria;

[4]Institute of Biosciences, Life Science Center, Vilnius University, Vilnius, Lithuania;

[5]BioTechMed-Graz, Graz, Austria.

Corresponding author
Vanessa Stadlbauer, MD, PhD
Department of Internal Medicine
Division of Gastroenterology and Hepatology
Medical University of Graz
Auenbruggerplatz 15
8036 Graz
Austria
Phone: 0043 316 385 82282
Email: *vanessa.stadlbauer@medunigraz.at*

SUPPLEMENTARY DATA

Calprotectin measurement in stool samples using a stabilizer solution

Calprotectin in stool was measured by ELISA (IDK® Calprotectin (MRP 8/14) (Stool) ELISA, K6927-230111, Immundiagnostik, Bensheim, Germany) according to the manufacturer´s instructions. The assay utilizes the two-site sandwich technique with two selected monoclonal antibodies that bind to human calprotectin. Stool samples were collected and stored in tubes containing a DNA/RNA stabilizer solution (Zymo Research, Irvine, CA, USA). 15 mg of the native stool samples were first diluted 1:100, in extraction buffer, then 40 µl supernatant was further diluted 1:25 in dilution buffer resulting in a 1:2500 dilution factor. 15 µl of the samples, diluted in 1:10 in stool stabilizer, were diluted 1:250 in buffer. Since first attempts to measure calprotectin in stool samples, stored in the stabilizer, yielded implausible low values, we performed a validation study in 10 stool samples of healthy volunteers.

The calprotectin levels were measured from native stool samples and stool samples stored in the stabilizer solution. Calprotectin levels were significantly lower in the stool samples stored in the stabilizer solution compared to native stool ($p = 0.011$). The recovery rate for calprotectin was 4.3 ± 4.8% compared to values measured in native stool samples. Bland-Altman analysis (Bias: 56.6, SD of bias: 57.3, Min: -55.7, Max: 168.9) showed that the difference between paired measurements increased with the average of paired measurements to an extent, which suggests that the values measured in the sample stored in the stabilizer solution did not exceed the background signal. The average of paired measurements was approximately half of the difference between the paired measurements, which indicates that only one of the measurements determined these parameters and the second measurement was close to zero (Figure S**1**). Calprotectin levels measured in the native samples and in the stabilizer did not correlate with each other (Pearson correlation: $r = 0.12$, $p = 0.74$).

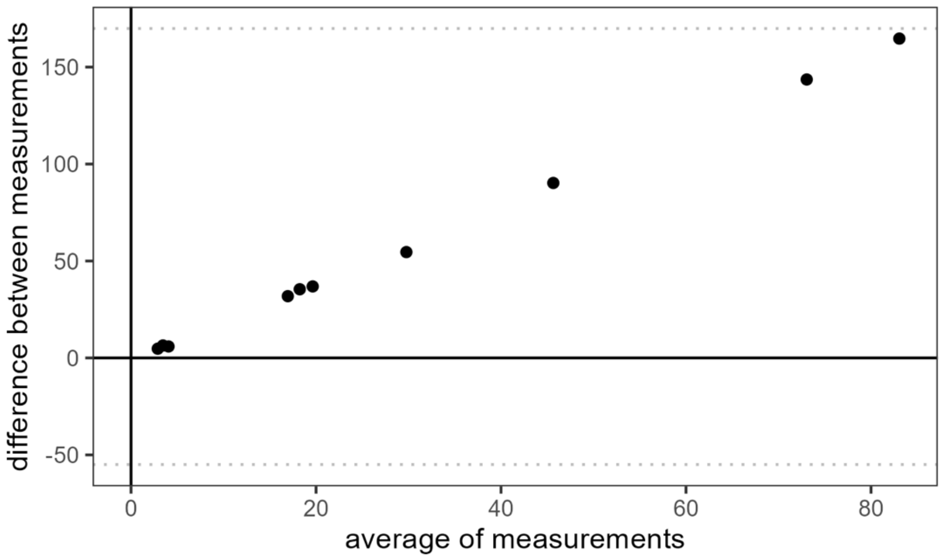

Figure S1: Bland-Altman plot of differences between measurements of calprotectin in native stool and in stool collected in the stabilizer.

Zonulin measurement in stool samples using a stabilizer solution

Zonulin in stool was measured by ELISA (IDK® Zonulin (Stool) ELISA, K5600-221201, Immundiagnostik, Bensheim, Germany) according to the manufacturer´s instructions. This assay is based on the method of competitive ELISA. Stool samples were collected and stored in tubes containing a DNA/RNA stabilizer solution (Zymo Research, Irvine, CA, USA). 15 mg of the native samples were diluted 1:50 in extraction buffer. 30 µl of the samples, diluted 1:10 in stool stabilizer, were diluted 1:5 in the kits dilution buffer, resulting a 1:50 dilution factor. Since first attempts to measure zonulin in stool samples stored in the stabilizer yielded implausible high values, we performed a validation study in 10 stool samples of healthy volunteers.

Zonulin levels from samples stored in stabilizing solution were higher than levels in native samples, however not significant ($p = 0.097$). The measured zonulin levels in stabilizing solution correlated negatively with the zonulin levels in native stool samples ($r = -0.10$, $p = 0.77$). As the measured zonulin levels in the stool samples stored in the stabilizing solution were high, but the agreement with the native samples was low, a serial dilution was performed clarify whether the stabilizing solution interfered with the measurement. When diluting the standard curve in the stabilizing solution high values were measured even when the protein concentration was low. This suggests that the stabilizing solution interfered with the measurement of the ELISA and leads to the high values measured in the samples.

In conclusion it was not possible to measure calprotectin or zonulin by ELISA in stool samples stored in DNA/RNA stabilizer solution (Zymo Research, Irvine, CA, USA).

SUPPLEMENTARY FIGURES

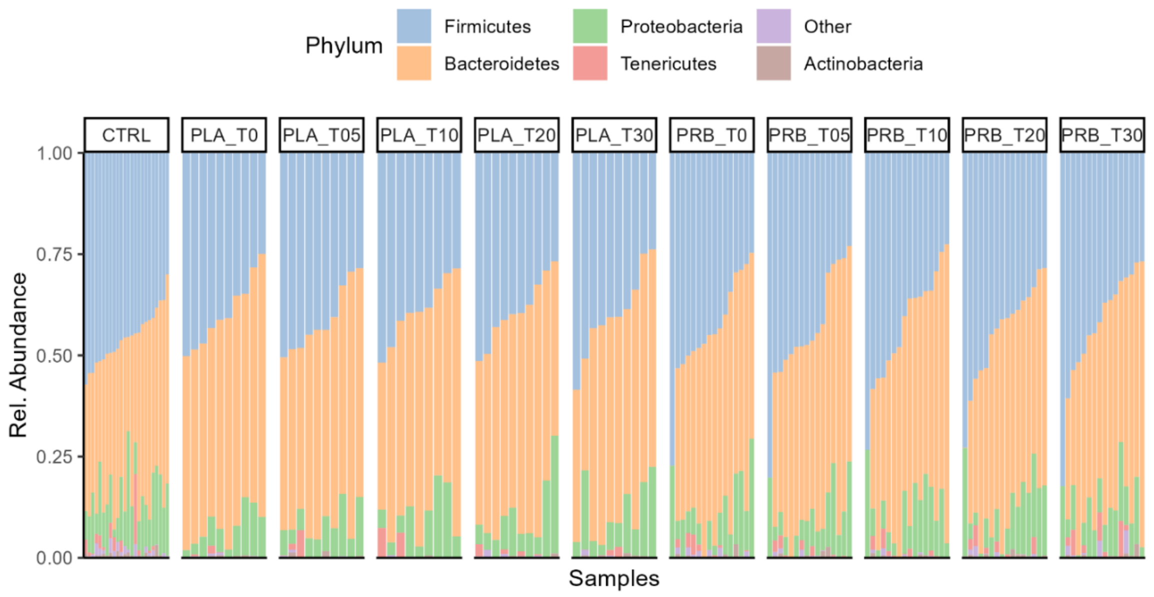

Figure S2: Top 5 phyla observed in the microbiome; CTRL - uninfected controls, PLA – Placebo, PRB - Probiotics

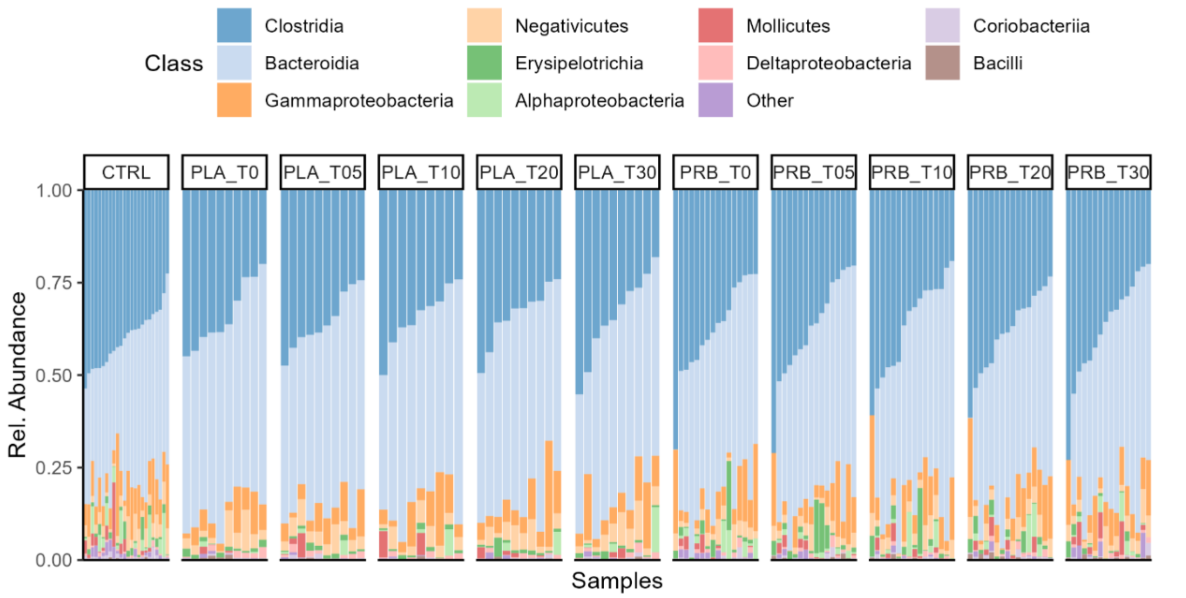

Figure S3: Top 10 classes observed in the microbiome; CTRL - uninfected controls, PLA – Placebo, PRB - Probiotics

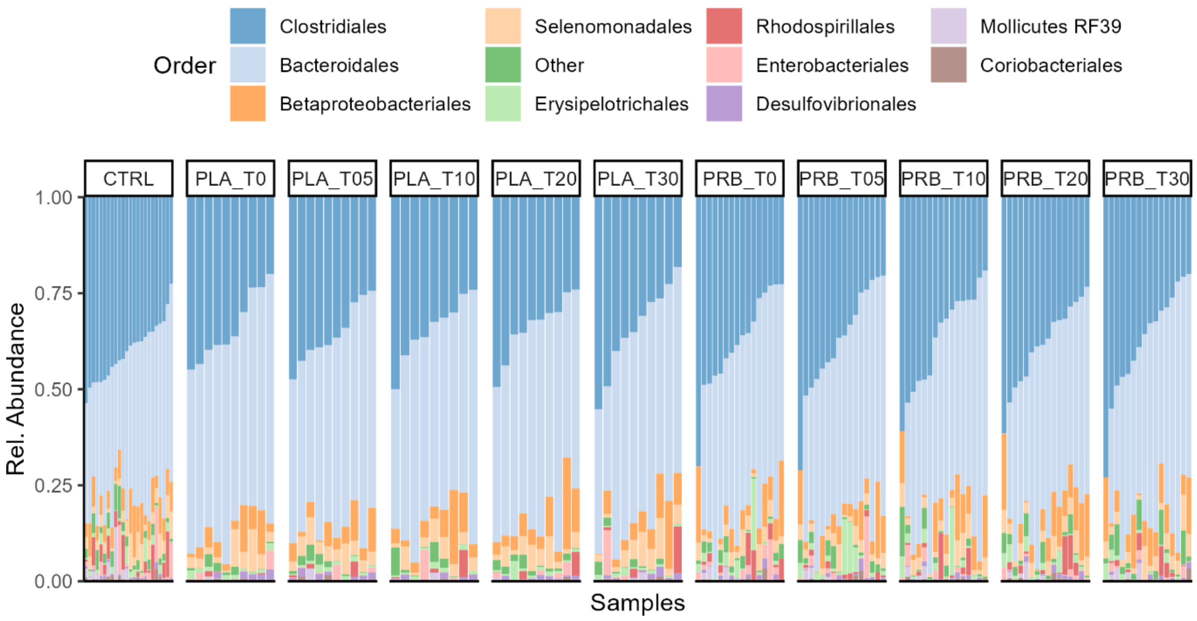

Figure S4: Top 10 orders observed in the microbiome; CTRL - uninfected controls, PLA – Placebo, PRB - Probiotics

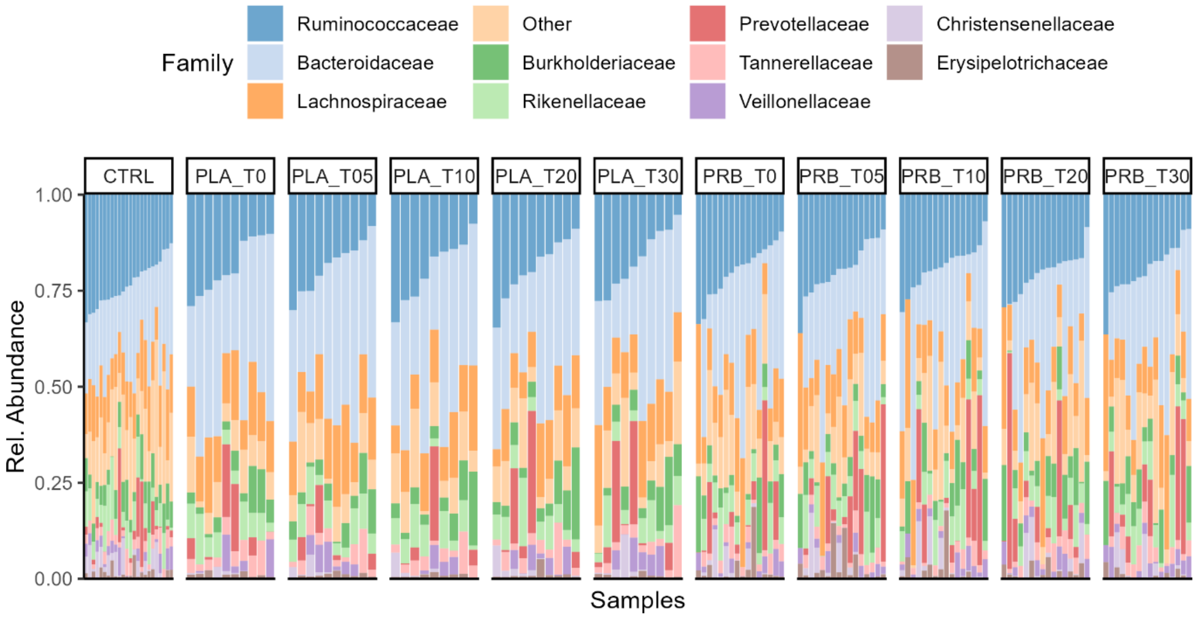

Figure S5: Top 10 families observed in the microbiome; CTRL - uninfected controls, PLA – Placebo, PRB - Probiotics

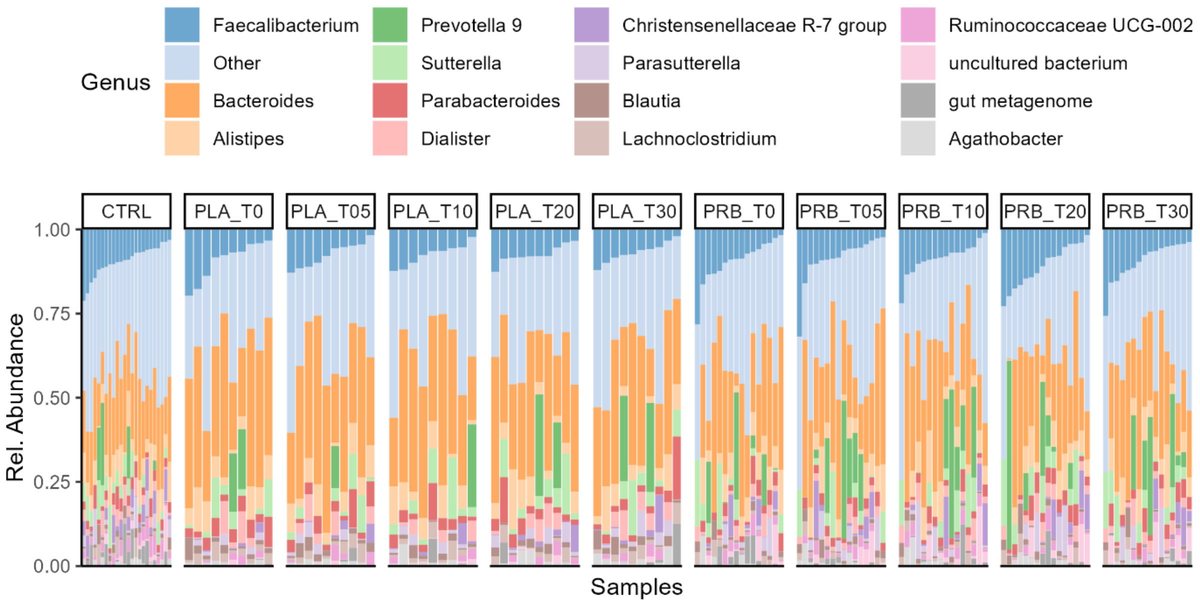

Figure S6: Top 15 genera observed in the microbiome; CTRL - uninfected controls, PLA – Placebo, PRB - Probiotics

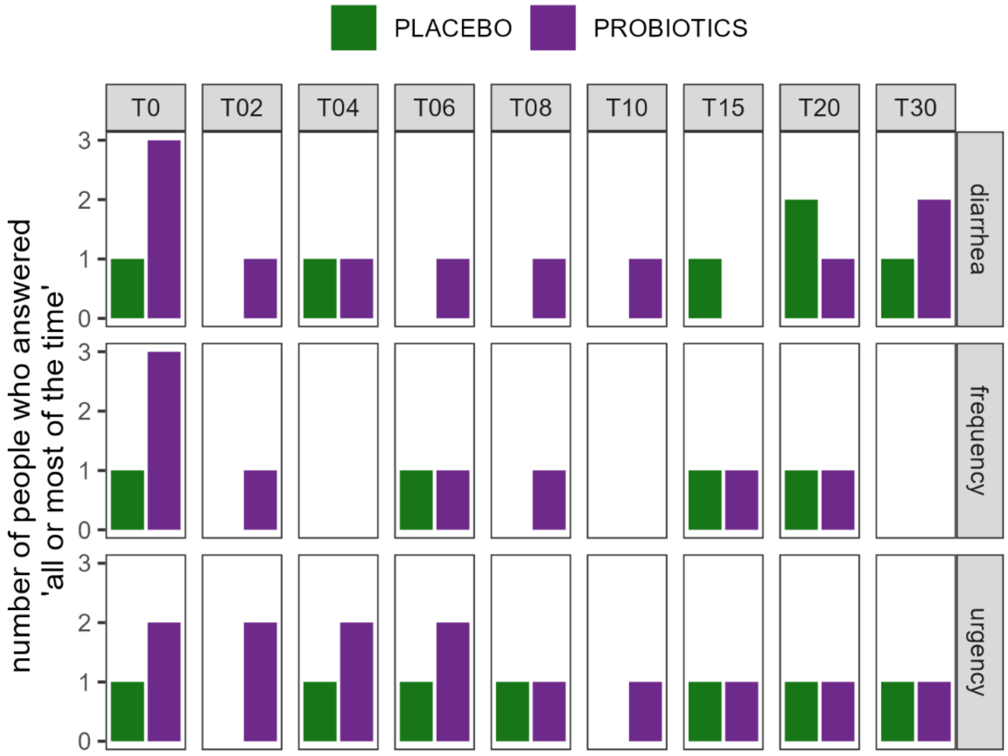

Figure S7: Results from the gastrointestinal quality of life index questions regarding diarrhea and the need for frequent or urgent bowel evacuations. Bar charts show the number of people answering the questions if they are inconvenienced by diarrhea (upper panel), frequent bowel movements (middle panel) or urgent bowel movements (lower panel) with “Yes, all the time” or “Yes, most of the time”, according to their group allocation.

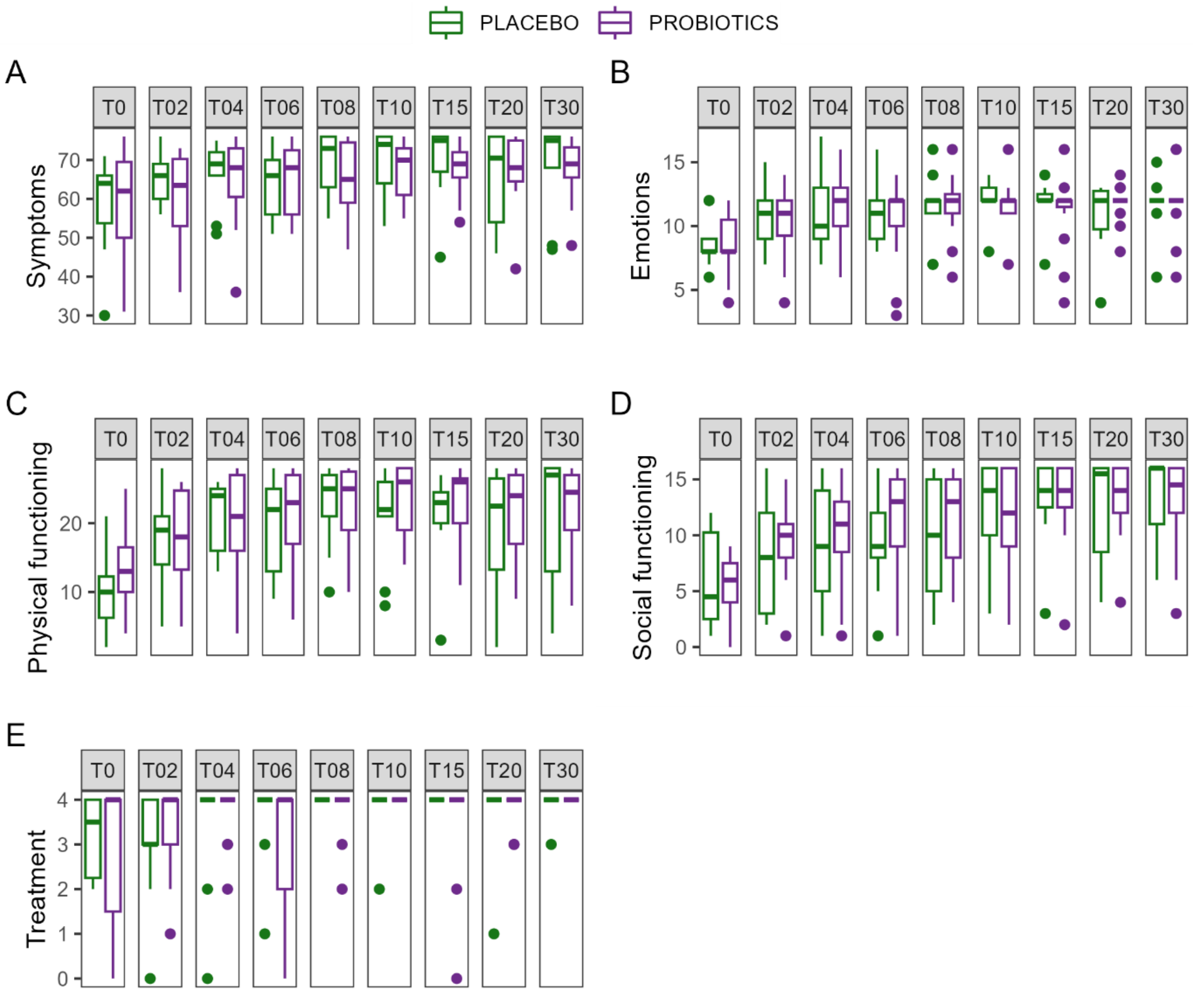

Figure S8: Development of the gastrointestinal quality of life during the study period in both treatment groups.

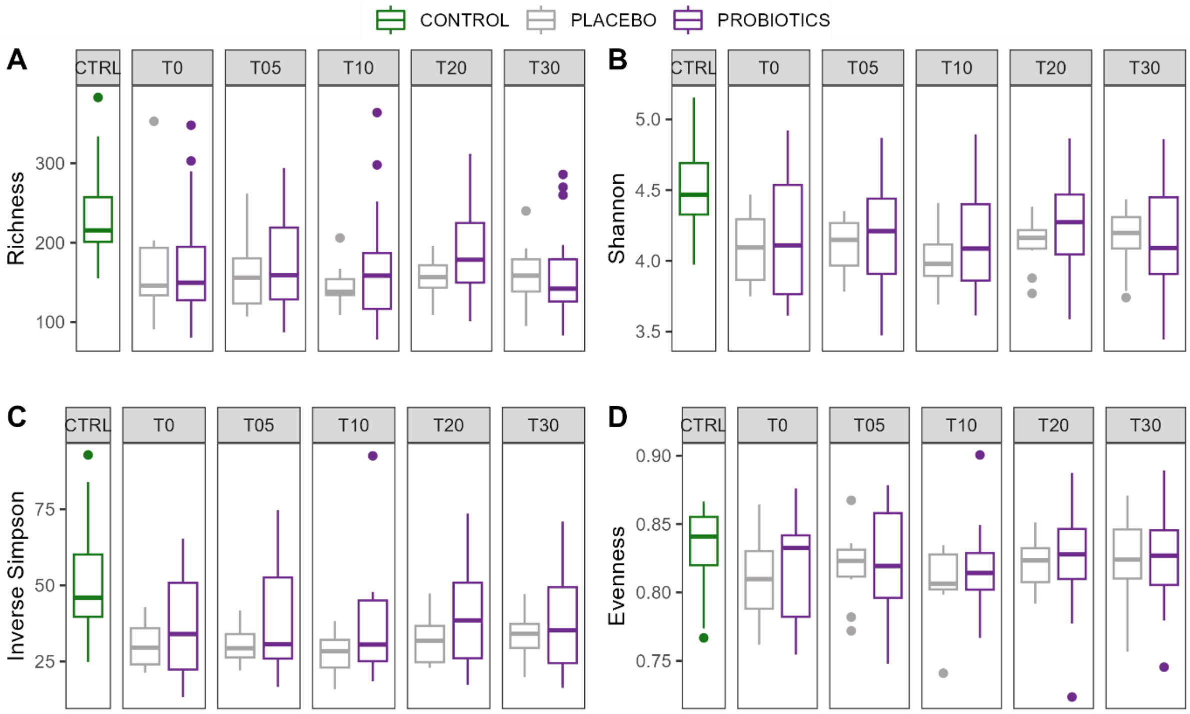

Figure S9: Alpha diversity metrics of patients with COVID19-infection throughout the study period and compared to healthy controls.

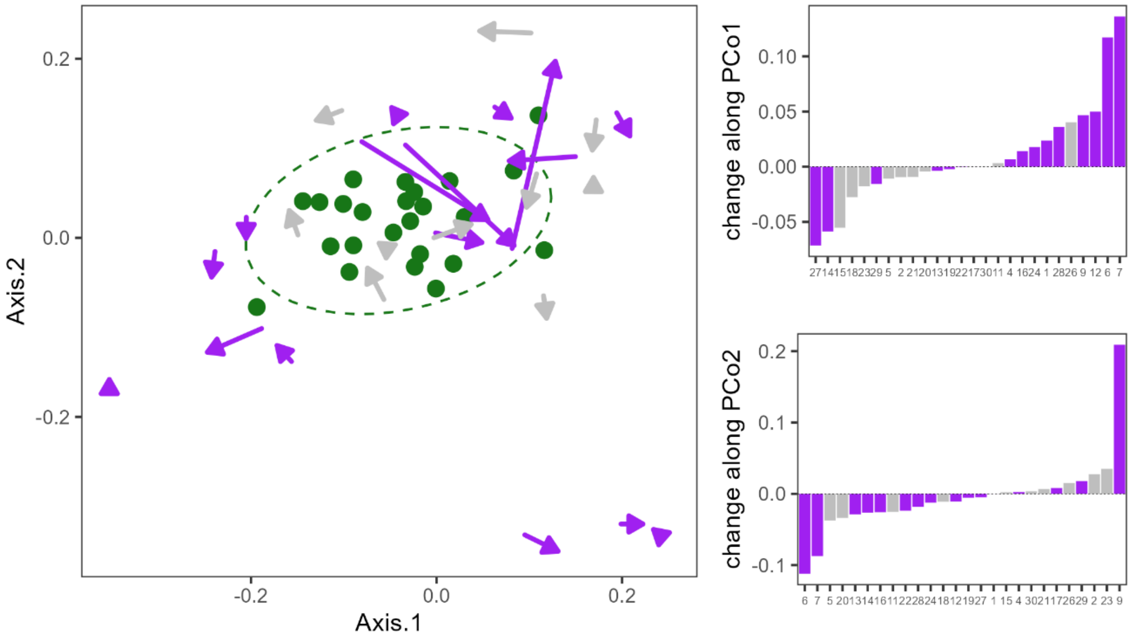

Figure S10: Similarity of the microbiome composition of healthy controls and patients with COVID19-infection before and at the end of intervention. Arrows represent the changes of a patient's microbiome from the beginning (base) and the end (tip) of the intervention, purple represents the probiotic group, grey the placebo group and green represents the non-infected controls.

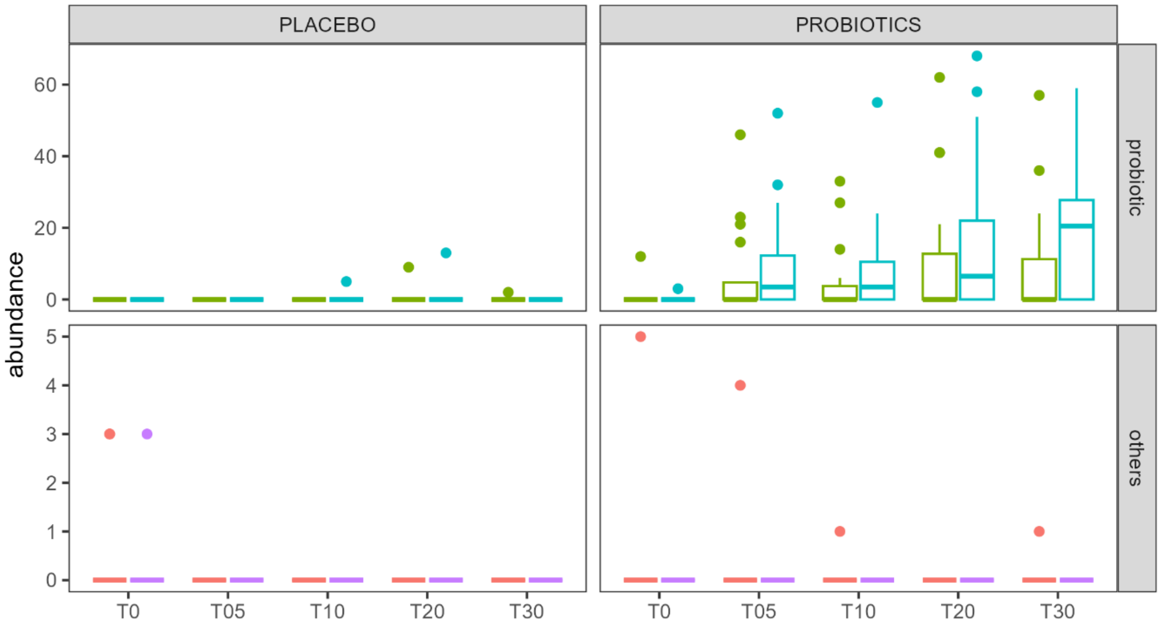

Figure S11: Enterococci ASVs found throughout the study according to timepoint and group allocation. Unique ASVs are presented in different colors. Blue and green bars represent the ASVs that most likely originated from the probiotic bacterium in the study product.

SUPPLEMENTARY TABLES

Table S1: Read counts before and after bioinformatics processing and quality filtering in all groups and at all timepoints.

**Description**	**Raw reads (mean)**	**High quality reads (mean)**
PLACEBO T0	30880 (± 17861)	19402 (±15284)
PLACEBO T05	29767 (± 11668)	17267 (8811)
PLACEBO T10	29516 (± 7733)	16698 (±4656)
PLACEBO T20	28391 (±8307)	15673 (±3931)
PLACEBO T30	28921 (±13773)	16241 (±6533)
PROBIOTICS T0	25145 (±4721)	14965 (± 4299)
PROBIOTICS T05	27432 (±6600)	16323 (± 5010)
PROBIOTICS T10	25643 (±9002)	15617 (±7039)
PROBIOTICS T20	31547 (±23068)	19643 (±14991)
PROBIOTICS T30	23832 (±5494)	14680 (±5521)

Table S2: Results of the mixed effect models testing the influence of time and intervention on the recovery from COVID-19 disease

	Intervention		Time		Intervention*Time		Intercept	
Item	Estimate	p-value	Estimate	p-value	Estimate	p-value	Estimate	p-value
Symptoms of the upper respiratory tract (ARTIQ)	0.318 (1.282)	0.806	-0.160 (0.032)	<0.001	0.007 (0.041)	0.869	5.107 (1.007)	<0.001
Symptoms of the lower respiratory tract (ARTIQ)	-0.160 (0.876)	0.856	-0.090 (0.020)	<0.001	0.010 (0.025)	0.703	2.902 (0.688)	<0.001
Psychological symptoms (ARTIQ)	-0.476 (0.704)	0.504	-0.045 (0.018)	0.011	-0.014 (0.022)	0.544	2.425 (0.553)	<0.001
Sleep issues (ARTIQ)	-0.269 (0.499)	0.593	-0.021 (0.016)	0.190	-0.005 (0.020)	0.593	1.698 (0.392)	<0.001
Need for medication (ARTIQ)	0.314 (0.412)	0.453	-0.022 (0.008)	<0.001	-0.013 (0.010)	0.193	0.864 (0.324)	0.013
Antitussives	1.188 (2.084)	0.569	-0.088 (0.073)	0.227	-0.093 (0.104)	0.373	-6.497 (2.574)	0.012
Antipyretics	-0.526 (1.510)	0.727	-0.301 (0.130)	0.020	0.167 (0.133)	0.209	-1.398 (1.201)	0.244
COPD/asthma inhalators/tablets	14.09 (12.60)	0.26	0.51 (0.41)	0.210	-0.80 (0.49)	0.100	-23.74 (14.09)	0.090
Muscle pain (ARTIQ)	-0.261 (0.194)	0.190	-0.017 (0.005)	0.001	0.007 (0.006)	0.253	0.609 (0.153)	<0.001
Tiredness (ARTIQ)	-0.121 (0.262)	0.647	-0.022 (0.007)	0.002	0.008 (0.008)	0.367	0.940 (0.206)	<0.001
Staying in bed (ARTIQ)	-0.191 (0.127)	0.140	-0.022 (0.006)	<0.001	0.004 (0.007)	0.524	0.557 (0.100)	<0.001
Cancelled work (ARTIQ)	-1.933 (1.195)	0.106	-0.180 (0.048)	<0.001	-0.002 (0.061)	0.973	2.507 (0.975)	0.010
Cancelled leisure activities (ARTIQ)	0.147 (1.638)	0.929	-0.167 (0.052)	0.001	-0.073 (0.070)	0.297	2.726 (1.337)	0.041

ARTIQ: Acute respiratory tract infection questionnaire.
